# Evaluation of the Healing Potential of Demineralized Dentin Matrix Fixed with Recombinant Human Bone Morphogenetic Protein-2 in Bone Grafts

**DOI:** 10.3390/ma10091049

**Published:** 2017-09-07

**Authors:** Sang-Yun Kim, Young-Kyun Kim, Yeoung-Hyun Park, Joo-Cheol Park, Jeong-Kui Ku, In-Woong Um, Ji-Yun Kim

**Affiliations:** 1 Department of Oral and Maxillofacial Surgery, Section of Dentistry, Seoul National University Bundang Hospital, 82 Gumi-ro 173, 173beon-gil, Bundang-gu, Seongnam 13620, Korea; 54248@snubh.org (S.-Y.K.); kujk123@gmail.com (J.-K.K.); 2 Department of Dentistry & Dental Research Institute, School of Dentistry, Seoul National University, Seoul 03080, Korea; pyh5436@snu.ac.kr (Y.-H.P.); jcapark@snu.ac.kr (J.-C.P.); 3 Department of Oral Histology-Developmental Biology, School of Dentistry, Seoul National University, Seoul 03080, Korea; 4 R&D Institute, Korea Tooth Bank, Seoul 06101, Korea; h-bmp@hanmail.net; 5 Department of Science Education, College of Education, Dankook University, Yongin 16890, Korea; kjy4549@naver.com

**Keywords:** rhBMP-2, demineralized dentin matrix

## Abstract

We aimed to evaluate the efficacy of demineralized dentin matrix (DDM) fixed with recombinant human bone morphogenetic protein-2 (rhBMP-2) through an experimental and a clinical study. Unilateral upper second and third premolars of eight beagles were extracted. A mucoperiosteal flap was elevated around the extraction socket, and a bone defect was made using a surgical drill. Each DDM was fixed with rhBMP-2, and autogenous bone was grafted at the bone defect area with a collagenous membrane. The beagles were euthanized at two, four, eight, and 12 weeks after receiving the bone graft. Block specimens involving grafted bone and surrounding natural bone were extracted. A total of 23 patients who received bone grafts using human DDM fixed with rhBMP-2 (AutoBT BMP) with implant placements (36 implants; maxilla: 14, mandible: 22) were selected. The implant stability, marginal bone loss, and clinical outcome were evaluated. Three trephine cores were harvested fourmonths after bone grafting, and histologic examination was performed. In the histological evaluation performed four weeks after the bone graft, autogenous bone showed 52% new bone formation and DDM fixed with rhBMP-2 showed 33% new bone formation. Twelve weeks after the bone graft, autogenous bone showed 75% new bone formation and DDM fixed with rhBMP-2 showed 48% new bone formation. In the clinical study, favorable osseointegration was obtained in 35 out of 36 implant sites (one case of osseointegration failure). In all cases, severe complications were not observed. Histomorphometrically, new bone formation was observed in 14.98% of the cases. The residual DDM particles were 6.22%. AutoBT BMP provides good osteoinductive and osteoconductive potential and clinical efficacy.

## 1. Introduction

Autogenous bone has osteogenic, osteoinductive, and osteoconductive abilities and is an ideal bone graft material for defects with increased susceptibility to infections and poor blood circulation. The major drawbacks of autogenous bone are that it requires a secondary donor site and can be collected in a rather limited amount. Allogenic bone graft, which was developed as an alternative material for autogenic bone graft, has osteoinductive ability, but facesthe risk of viral infections, while heterogenic bone and synthetic bones have only osteoconductive ability; therefore, these grafts are limited in their ability to promote the regeneration of new bone [1]. The teeth are known to be an organic-inorganic hybrid composed of calcium phosphates with collagen and other organic compounds. The minerals of the teeth consist of fivetypes of biological calcium phosphate (hydroxyapatite (HA), tricalcium phosphate (TCP), octacalcium phosphate (OCP), amorphous calcium phosphate (ACP), and dicalcium phosphate dehydrate (DCPD)). The fivetypes of calcium phosphate interact, and when implanted into the body, they are expected to produce good bone remodeling. The apatite in bone tissue is a polymer-ceramic nanohybrid. The apatite in human bone tissue has a low-crystalline structure with a particle size in the range of 10–100 nm. However, when HA is manufactured by sintering at a high temperature, it shows a high-crystalline structure and the sintering process leads to grain growth, resulting in a size at least an order of magnitude larger than the apatite in bone tissue. High crystallinity and large particle size make biodegradation almost impossible; bone turnover is very low and the HA cannot be broken down by osteoclasts. For these reasons, carbonic apatite, which has low crystallinity, shows the best bone turnover. Bones and teeth have a very similar chemical composition. Enamel consists of 96% inorganic substances and 4% organic substances and water; dentin consists of 65% inorganic substances and 35% organic substances and water; cementum consists of 45–50% inorganic substances and 50–55% organic substances and water; and alveolar bone consists of 65% inorganic substances and 35% organic substances and water. Cementum and dentin in the teeth contain a large quantity of bone growth factors, including type I collagen and bone morphogenetic proteins (BMPs). Hence, it is thought that the teeth could be used to develop bone graft materials that have ahealing ability similar to autogenous bone [2,3,4]. Research on graft materials that can overcome the limitations of autogenous bone and other bony substitutes has led to the development of autogenous demineralized dentin matrix (ADDM), which is an ideal substitute for autogenous bone. The ADDM has excellent osteoinductive and osteoconductive abilities, and the recovery process induced by ADDM histologically resembles that induced by autogenous bone [5,6,7,8].

BMPs bound to a scaffold have been used for sinus bone graft surgery and guided bone regeneration (GBR) in the field of dentistry. Various kinds of BMPs are used for additional osteoinductive effects when using bone graft materials that only have osteoconductive potential, and BMP-2, 4, 7, and 14 are known to have excellent osteoinductive effects. BMP-2 acts as a growth and differentiation factor within the body, exerts a wide range of effects during osteogenesis in which mesenchymal stem cells differentiate into osteoblasts, and promotes new bone formation [9,10,11,12,13,14]. Studies showed clinically-satisfactory outcomes of bone graft surgeries using a recombinant human bone morphogenetic protein-2 (rhBMP-2) bound to a DDM powder and hydroxyapatite (HA) scaffold that was developed in Korea [15,16].

We performed two types of studies to assess the healing potential of DDM fixed with rhBMP-2. First, we performed an animal experiment in which human DDM fixed with rhBMP-2 and autogenous bone grafts were grafted onto calvarial bone defects of beagles, and the recovery processes were compared. Second, we conducted a clinical study on the use of ADDM fixed with rhBMP-2 during bone graft to assess whether DDM can act as an ideal carrier of various growth factors, such as BMP.

## 2. Materials and Methods

### 2.1. Human DDM Fixed with rhBMP-2 Fabrication (Dip-Dry Method)

DDM powder was produced using human teeth according to the Korea tooth bank fabrication protocol. Extracted human teeth were soaked in 70% ethyl alcohol. After dividing the teeth into the crown and root, the root portion was collected and prepared for partially isolating DDM. The root was crushed to a powder. The size of the particles was between 200 and 1000 μm in diameter. Contaminants and the remaining soft tissues were removed by washing the crushed tooth powder. The crushed particles were soaked in distilled water and a hydrogen dioxide solution, and the remaining foreign substances were removed by using an ultrasonic cleaner. The cleaned particles were dehydrated with ethyl alcohol and went through defatting using ethyl ether solutions. The particles were then demineralized for 30 min in 0.6 N HCl. The demineralized particles were lyophilized and sterilized with ethylene oxide gas.

**Figure 1:**
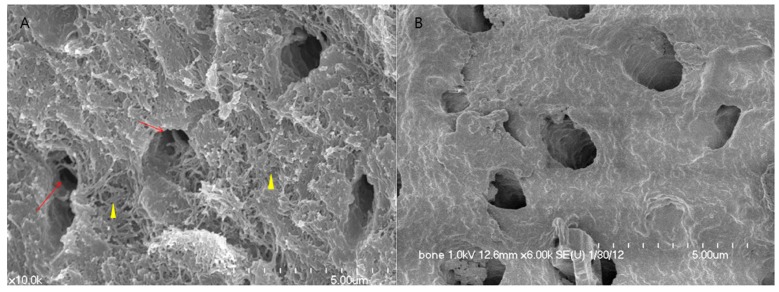
SEM image of DDM. (**A**) Surface of DDM. Cross-sectioned tubules showing exposed outer peritubular (arrow) and intertubular collagen fibrils (arrowhead) after demineralization; (**B**) Surface of DDM/rhBMP-2. rhBMP-2 (0.2 mg/mL, Cowell BMP, Busan, Korea) was fixed to DDM. Structurally, the exposed collagen fibrils are impregnated with the protein solution around the dentinal tubules and collagen matrix.

The rhBMP-2 was loaded on to the DDM powder by placing 5.0 μg of 0.2 mg/mL rhBMP-2 (Cowellmedi, Busan, Korea) and 0.03 g of DDM powder into individual 15-mL conical tubes. The mixtures were frozen in a deep freeze at −70 °C for 60 min, slotted into a lyophilization glass bottle, and then fixed in a lyophilizer (ILShin Lab, Seoul, Korea) (Figure 1). After sterilization with ethylene oxide, the DDM powder fixed with rhBMP-2 was packed and transported to the hospital where the implant surgery would be performed [16]. For scanning electron microscopy (SEM), samples were fixed in 0.1 M cacodylate buffer (pH 7.3) containing 2.5% glutaraldehyde for 30 min and in 0.1 M cacodylate buffer (pH 7.4) containing 1% osmium tetroxide for 1 h. After rapid dehydration through an ethanol gradient, critical point drying, and sputter-coating with gold, cells were observed under SEM (S-4700, HITACHI, Tokyo, Japan) (Figure 1).

### 2.2. Animal Experimental Study: Assessment of Bone Recovery after Placement of Human DDM Fixed with rhBMP-2 and Autogenous Bone Grafts in Beagles

**Figure 2:**
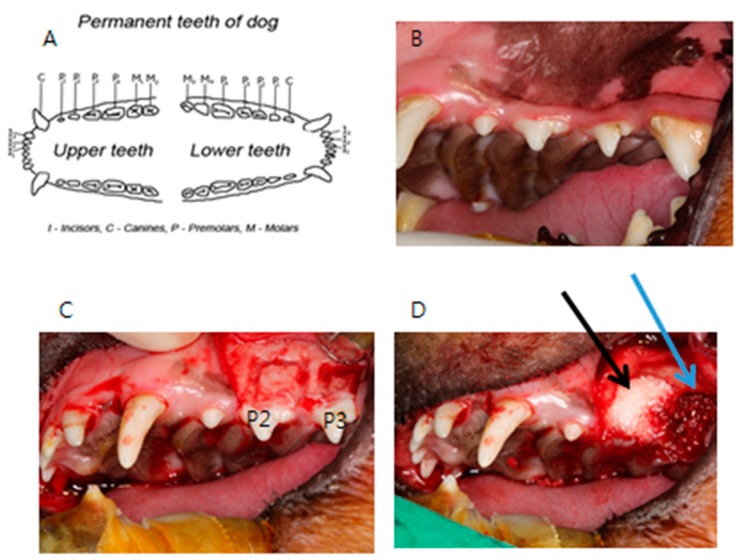
Photograph of the animal experiment. (**A**) Permanent teeth of the beagle; (**B**) Intraoral photograph of the beagle; (**C**) Unilateral maxillary second (P2) and third premolars (P3) were extracted and mucoperiosteal flaps were elevated. The rectangular bone defects measuring 5 mm × 8 mm on the second and third premolars were made; (**D**) Autogenous bone was grafted onto one of two bone defects (control group, blue arrow), and human DDM fixed with rhBMP-2 was grafted onto the other (experimental group, black arrow).

This animal study was performed under the approval of Institutional Animal Care and Use Committee (IACUC), Seoul National University Bundang Hospital (BA1509-185/064-01). To compare the recovery processes between human DDM fixed with rhBMP-2 and autogenous bone, a total of eight beagles were used in the experiment. After the oral cavities of the animals were sterilized, a sterile drape was placed to isolate the oral cavities, and minimally-invasive anesthesia using 2% lidocaine HCL (containing only 1:10 of epinephrine) was administered to minimize bleeding. Unilateral maxillary second and third molars were extracted with an extraction forceps, elevator, and surgical drill. Mucoperiosteal flaps were elevated around the extraction sockets, and a surgical drill was used to form rectangular bone defects measuring 5 mm × 8 mm on the second and third premolars. The palatal cortical bone was not removed and was left intact. Autogenous bone collected during the process of extraction and bone defect formation was wrapped in gauze soaked in a saline solution for later use. Autogenous bone was grafted onto one of two bone defects (control group), and human DDM fixed with rhBMP-2 was grafted onto the other (experimental group). Both defects were covered with an absorbable collagen membrane (OSSIX^®^ PLUS, Dantum Dental Ltd., 1 Bat Sheva St., Telrad Industrial Park, Israel) and sutured (Figure 2).

Two beagles were sacrificed at two, four, eight, and 12 weeks after bone grafting, and block samples containing the grafted bone and the surrounding bone tissues were collected. The samples were demineralized in 10% formic acid (Georgiachem Inc., Atlanta, GA, USA) for three weeks, subjected to tissue processing in a microtome (Shadon Citadel 2000, Thermo Fisher Scientific Inc., Kalamazoo, MI, USA), and embedded on paraffin blocks (Shadon Histocentre 3, Thermo Fisher Scientific Inc., Kalmazoo, MI, USA). Next, all samples were continuously sectioned into 5-μm sections with a microtome (Shadon Finesse 325, Thermo Fisher Scientific Inc., Kalamazoo, MI, USA), and were subjected to hematoxylin and eosin (H & E) staining and Masson’s trichrome staining. For immunohistochemistry, proteins were detected with anti-DMP1 (Santa Cruz Biotechnology, Dallas, TX, USA) or anti-BSP (Santa Cruz Biotechnology) at a dilution of 1:100 as the primary antibody and a biotin-labeled goat anti-rabbit IgG (Vector Labs, Burlingame, CA, USA) as the secondary antibody. For histomorphometric analysis, an optical microscope (BX51, Olympus Co., Tokyo, Japan) connected to a computer and charge-coupled device (CCD) camera (SPOT Insight 2Mp scientific CCD digital camera system, Diagnostic Instruments Inc., Sterling Heights, MI, USA) and an adaptor (U-CMA3, Olympus Co., Tokyo, Japan) was used to take images of the samples. Image analysis was performed with SPOT Software V4.0 (Diagnostic Instruments Inc., USA). New bone formation was observed using Pro Plus^®^ (Media Cybernetics Inc., Warrendale, PA, USA) analysis software.

### 2.3. Human Clinical Study and a Case Report: Clinical Study of a Variety of Bone Graft Using ADDM Fixed with rhBMP-2

The teeth extracted from the patients were used to produce ADDM fixed with rhBMP-2, which was later used in bone graft surgery. The product name of the material is AutoBT BMP (Korea Tooth Bank, Seoul, Korea). We conducted a prospective observational study by including patients who underwent bone grafting and implant placement using AutoBT BMP between May 2015 and January 2016 at Seoul National University Bundang Hospital. The present study was conducted in accordance with the Declaration of Helsinki, and the protocol was approved by the Ethics Committee of Seoul National University of Bundang Hospital (B-1441/274-106).

Patient selection criteria were as follows:

1. Patients requiring a bone graft at the site of implant placement;
2. Patients whose teeth could be extracted and used to make ADDM fixed with rhBMP-2 for use as bone graft material;
3. Patients who consented to the clinical study;
4. Patients who were healthy or had controlled systemic disease; and
5. Non-smokers.

A total of 23 patients were included in this study, with a mean age of 59.2 ± 13.1 years (range 39–82 years). There were 16 men and seven women. A total of 36 implants (14 in the maxilla and 22 in the mandible) were placed. Types of bone graft surgeries performed included GBR, sinus bone grafting, and ridge augmentation. Thirty-three implants were placed at the same time as bone grafting, and three implants were placed 4–5 months after bone grafting (Table S1).

The types of bone grafting performed, complications related to the surgery, and marginal bone loss around the implants were investigated. The primary and secondary implant stabilities were measured using Osstell^®^ Mentor (Osstell, Göteberg, Sweden). Primary stability was measured immediately after implant placement, and secondary stability was measured during a secondary surgery or impression-taking. Digital periapical radiographs of marginal bone loss around the implants were obtained by using the paralleling technique. The radiographs obtained immediately after implant placement were set as the baseline and were compared with radiographs obtained during the last follow-up. Magnification was determined by calculating the ratio of the length of the actual implant fixture to the length of the fixture on periapical radiographs, and the mean amounts of alveolar bone absorption on the mesial and distal areas of the implants were calculated. For the radiographic measurements, the IMPAX system software program (Agfa-Gevaert Group, Mortsel, Belgium) was used. In addition, tissue samples were collected from three patients who underwent bone grafting and delayed implant placement after obtaining the patients’ consent. The three specimens were demineralized in 10% formic acid for 14 days and then embedded in paraffin. Serial sections of 5-μm thickness were obtained through the horizontal plane of the vertical alveolar defects, from which, two sections that contained the whole defect were selected and stained with H and E. In a histomorphometric analysis, an optical microscope (BX51, Olympus Co., Tokyo, Japan) connected to a computer and charge-coupled device (CCD) camera (SPOT Insight 2 megapixel scientific CCD digital camera system, Diagnostic Instruments Inc., Sterling Heights, MI, USA), and an adaptor (U-CMA3, Olympus Co., Tokyo, Japan) was used to take images of the samples. Image analysis was performed with SPOT Software V4.0 (Diagnostic Instruments Inc., Burroughs, Sterling Heights, MI, USA). The ratio of newly-formed bone area, residual dentin, and soft tissue component area compared to total area were observed using Pro Plus^®^ (Media Cybernetics Inc., Warrendale, PA, USA) analysis software by an examiner who was unaware of the type of grafted material.

## 3. Results

### 3.1. Animal Experimental Study

#### 3.1.1. Histologic Findings

**Figure 3:**
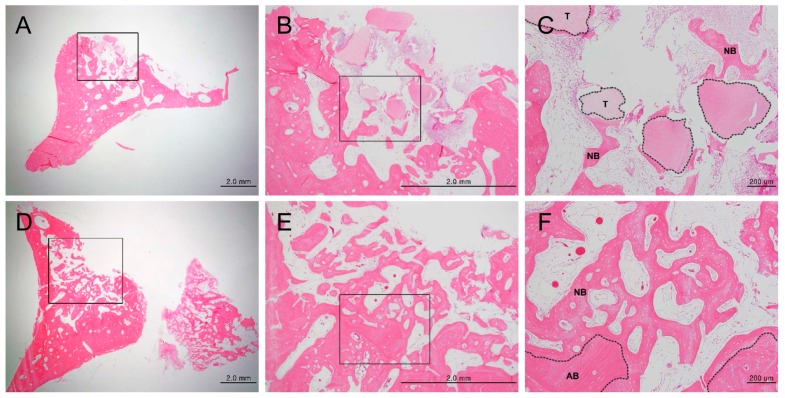
Histological analysis of new bone formation at the bone defect area after four weeks, by hematoxylin/eosin staining (magnification: (**A**,**D**) 12.5×; (**B**,**E**) 40×; (**C**,**F**) 100×). (**A**–**C**) Experimental group (DDM fixed with rhBMP-2); (**D**–**F**) Control group (autogenous bone graft). Figures (**B**) and (**E**) are higher magnification views of figures (**A**) and (**D**), respectively. Figures (**C**) and (**F**) are higher magnification views of figures (**B**) and (**E**), respectively. (**C**): The area enclosed within the dotted line represents DDM fixed with rhBMP-2, and newly-formed bone seen around the DDM. (**F**) The area within the dotted line indicates autogenous bone graft, and newly-formed bone found around the autogenous bone graft. Scale bars: 2.0 mm (**A**,**B**,**D**,**E**), 200 μm (**C**,**F**). T: DDM fixed with rhBMP-2, NB: new bone, AB: autogenous bone.

**Figure 4:**
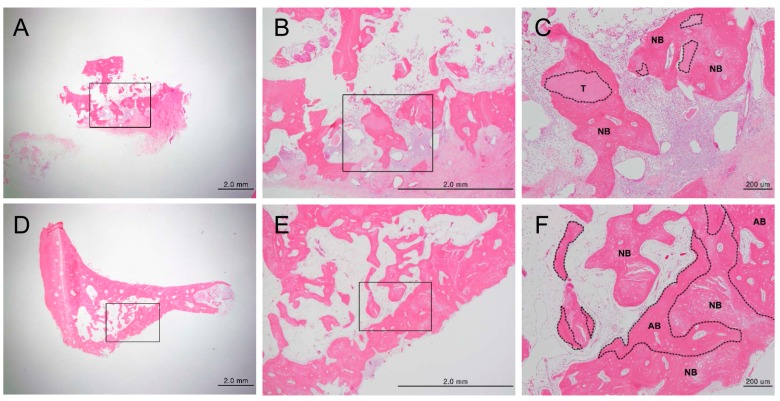
Histological analysis of new bone formation at the bone defect area after eight weeks, by hematoxylin/eosin staining (magnification: (**A**,**D**) 12.5×; (**B**,**E**) 40×; (**C**,**F**) 100×). (**A**–**C**) Experimental group (DDM fixed with rhBMP-2); (**D**–**F**) Control group (autogenous bone graft). Figures (**B**) and (**E**) are higher magnification views of figures (**A**) and (**D**), respectively. Figures (**C**) and (**F**) are higher magnification views of figures (**B**) and (**E**), respectively. (**C**) The area enclosed within the dotted line represents DDM fixed with rhBMP-2, and newly-formed bone seen around the DDM. (**F**) The area within the dotted line indicates the autogenous bone graft, and newly-formed bone found around the autogenous bone graft. Scale bars: 2.0 mm (**A**,**B**,**D**,**E**), 200 μm (**D**,**F**). T: DDM fixed with rhBMP-2, NB: new bone, AB: autogenous bone**.**

**Figure 5:**
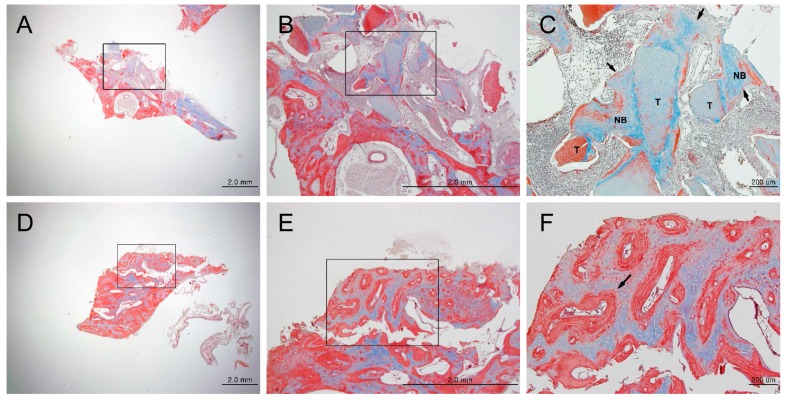
Masson-trichrome staining in each group showing findings for new bone formation after eight weeks (magnification: (**A**,**D**) 12.5×; (**B**,**E**) 40×; (**C**,**F**) 100×). (**A**–**C**) Experimental group (DDM fixed with rhBMP-2); (**D**–**F**) Control group (autogenous bone graft). Figures (**B**) and (**E**) are higher magnification views of figures (**A**) and (**D**), respectively. Figures (**C**) and (**F**) are higher magnification views of figures (**B**) and (**E**), respectively. (**C**) General woven bone (blue color) formed around the TCP and mineralized bone (red color) was seen (arrow). (**F**) The amount of mineralized bone (red color) was greater in the autogenous bone graft group (arrow).

**Figure 6:**
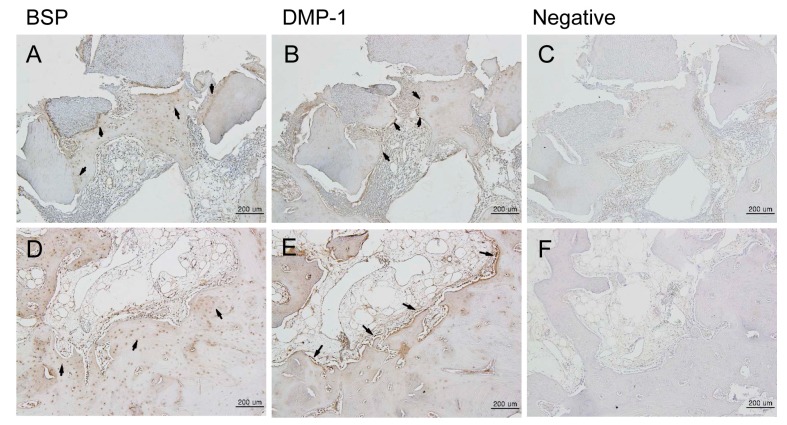
Immunohistochemical analysis of new bone formation at the bone defect area after four weeks (100×). BSP, DMP-1 proteins were expressed in the area of new bone formation after four weeks. (**A**–**C**) Experimental group (DDM fixed with rhBMP-2); (**D**–**F**) Control group (autogenous bone graft). New bone tissues were immunostained with anti-BSP (**A**,**D**) and anti-DMP-1 (**B**,**E**). Negative controls (**C**,**F**) included no primary antibody. (**A**,**D**) BSP expression was detected by immunohistochemistry in the area of new bone formation. BSP was expressed by osteocytes (arrows). (**B**,**E**) DMP-1 was expressed by osteoblasts (arrows). Scale bars: 200 μm.

H and E staining showed new bone formation starting at two weeks after bone grafting, which increased over time, as well as maturation and calcification of the new bone. In the experimental group, new bone formation was observed around the implants (Supplementary Materials Figure S1A–C). In the control group, new bone formation was observed at the margins of the bone defects (Figure S1D–F). In the experimental group, osteogenesis began with the differentiation of mesenchymal cells into osteoblasts, which then surrounded the implants (Figure S1C). Active new bone formation was observed at four weeks after bone grafting, in both groups. Osteoblasts were well-arranged around the newly-formed bone (Figure 3). Increased new bone formation was observed at eight weeks after bone grafting, in both groups. In the experimental group, new bone, which was now mature, was observed surrounding the implants (Figure 4A–C). In the control group, new bone was observed to form along one side of the implants, and develop into typical lamellar bone (Figure 4D–F). New bone formation was observed to increase even further at 12 weeks after bone grafting, in both the groups. In the experimental group, new bone maturation had progressed more than in the control group, and new bone could no longer be differentiated from the existing bone (Figure S2A–C). A large amount of mature new bone was observed surrounding the implants in the control group (Figure S2D–F). Masson’s trichrome staining showed patterns of new bone formation in which osteoblasts surrounded the implants and induced bone formation. The amount of new bone increased from two weeks to 12 weeks after bone grafting. In the control group, osteoblasts were highly active starting at two weeks, and the amount of new bone significantly increased from two to 12 weeks after bone grafting. In the experimental group, the activity of the osteoblasts was maximum at eight weeks, and new bone that formed around the implants, or typical woven bone, was clearly observed. Newly-formed bone was significantly more mineralized in the control group than in the experimental group at eight weeks after bone grafting (Figure 5). Between four and eight weeks after bone grafting, osteocytes within the mature bone showed BSP-immunoreactivity, and osteoblasts that lined the new bone showed DMP1-immunoreactivity (Figure 6).

#### 3.1.2. Histomorphometric Analysis

**Figure 7:**
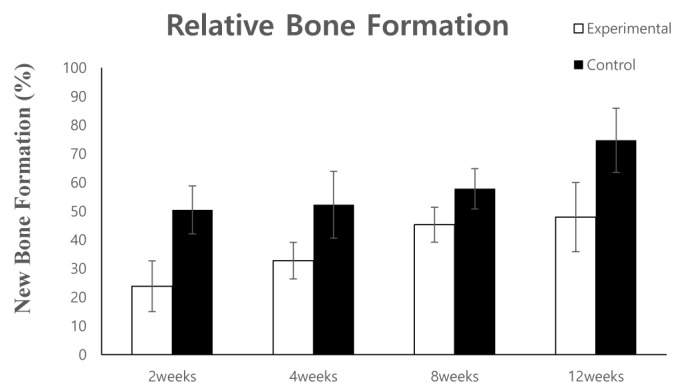
Histomorphometric analysis of new bone formation at the bone defect area.

New bone formation at 50.5 ± 8.4% and 23.9 ± 8.8%, at two weeks after bone grafting, and 52.2 ± 11.6%, and 32.8 ± 6.4% at four weeks after bone grafting, were observed in the control and experimental groups, respectively. At eight weeks after bone grafting, bone formation at 57.8 ± 7%, and 45.3 ± 6% was observed in the control and experimental groups, respectively. New bone formation was further increased at 12 weeks after bone grafting; new bone formation at 74.7 ± 11.2% and 48 ± 12.1% was observed in the control and experimental groups, respectively (Figure 7).

The area of new bone formed after grafting with the DDM fixed with rhBMP-2 and autogenous bone was measured at two, four, eight, and 12 weeks.

### 3.2. Human Clinical Study and Case Report

#### 3.2.1. Clinical Study

The mean length of follow-up after bone grafting was 10.5 months (range 3.1–17.8 months), for the 36 implants. The mean implant stability quotient (ISQ) for primary implant stability was 71.8, and that for secondary stability was 78.0; both were in the stable range. Marginal bone loss ranged from 0 mm to 0.8 mm, and was 0.4 mm on average (Table 1). Of the 36 implants, one demonstrated failure of initial osseointegration. No unusual complications were observed for any of the implants. Analysis of the three tissue samples that had been grafted into the maxilla before implant placement showed dense fibrous connective tissues and angiogenesis around the newly-formed bone. Histomorphometric analysis showed 14.98 ± 10.09% newly-formed bone, 6.22 ± 5.5% residual DDM, and 60.86 ± 18.66% soft tissue components in the tissues.

**Figure 8:**
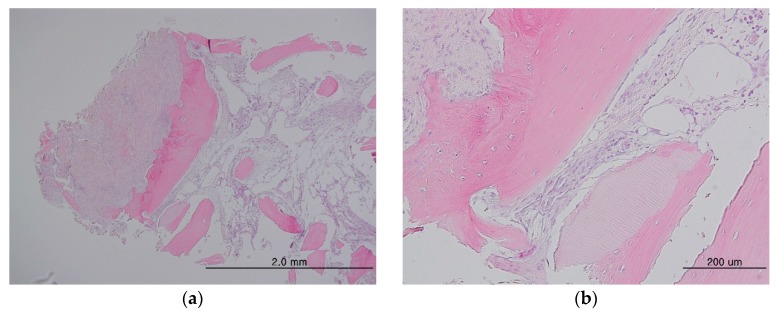
Histology specimens from the maxilla and sinus (**a**) showing dense fibrous connective tissue on the left of the newly-formed bone, whereas tissue rich in angiogenesis can be seen on the other side of the new bone. Higher magnification showing newly-formed bone with embedded osteocytes surrounding the particles and direct contacts between the bone and particle with the vasculature (**b**). No inflammatory cellular infiltration can be observed.

Histological specimens showed newly-formed bone associated with connective tissue stroma, rich in angiogenesis, in both groups. No inflammatory cellular infiltration was observed. DDM particles were surrounded by osteoid, indicating new bone formation and direct contacts were observed in both groups, demonstrating osteoconductive and osteoinductive properties of the DDM-based materials (Figure 8).

#### 3.2.2. Case Report

**Figure 9:**
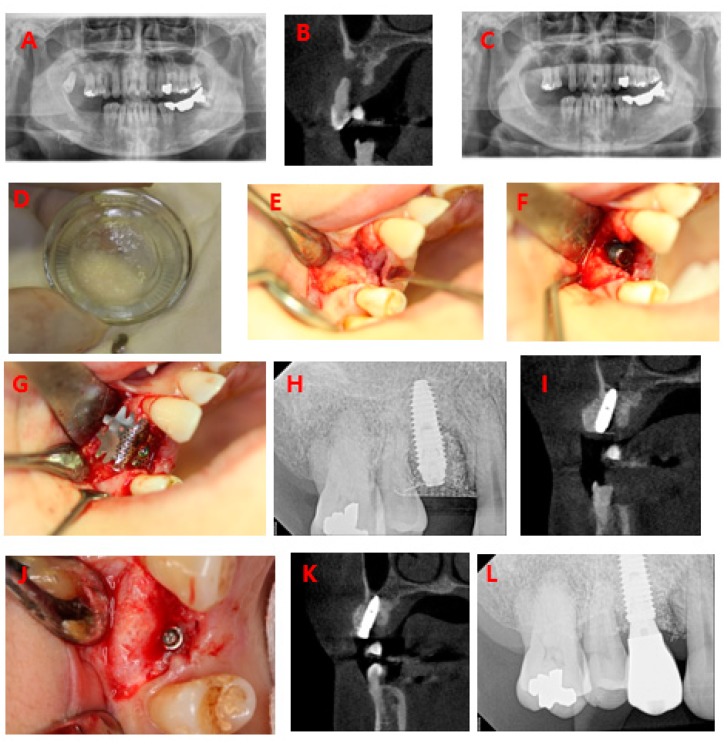
GBR case using AutoBT BMP. (**A**) Initial panoramic radiograph; (**B**) Initial CT view: severe alveolar bone resorption in the region of 14; (**C**) Panoramic radiograph after tooth extraction; (**D**) AutoBT; (**E**–**G**): First surgery in the region of 14; (**E**): Mucoperiosteal flap elevation; (**F**): Implant insertion and bone graft; (**G**) Titanium mesh covering; (**H**): Periapical radiograph after implantation; (**I**) CT view after implantation; (**J**) Second surgery; (**K**) CT view after second surgery; (**L**) Periapical radiograph after prosthetic loading.

A 54-year-old female patient visited our clinic with a chief complaint of multiple tooth loss and tooth mobility. Severe mobility of 14 and 18 and loss of 17, 46, and 47 were observed (Figure 9A,B). Accordingly, 14 and 18 were extracted and processed with AutoBT BMP(Korea Tooth Bank, Seoul, Korea), and bone grafting and implant placement in the region of 14 and 46 were planned (Figure 9C). AutoBT BMP was produced via fixation of rhBMP-2 (dip-dry method) at the Korea Tooth Bank (Figure 9D). The initial ISQs of 46 and 14 after implant placement were 83 and 52, respectively (Figure 9E–I). The second surgery was performed four months after implant placement. The ISQs for 46 and 14 at the time of the second surgery were 80 and 70, respectively; the implants were stable (Figure 9J,K). The patients were monitored for 1.5 years after the surgery. All implants were well-maintained without any complications (Figure 9L).

## 4. Discussion

BMPs with osteoinductive potential must be extracted from a tooth or hard tissue; however, the amount of BMPs that can be collected is limited, which subsequently limits their clinical application. Nevertheless, advances in genetic engineering have enabled mass production of rhBMP-2 through genetic recombinant technology using genes derived from *Escherichia coli* or mammalian cells, which are widely used in clinical settings [17,18,19,20,21]. Due to their water-soluble nature, BMPs are rapidly dissolved, and their osteoinductive ability is not utilized to its full potential when used alone. Therefore, a scaffold that can contain BMPs and adequately release them is necessary. While various materials have been used to develop scaffolds that can carry BMPs, they have presented problems, such as rapid absorption and BMP seeding, showing poor clinical results. Unlike other scaffolds, DDM has a microporous structure with dentinal tubules 1.2–2.5 μm in diameter, which allows it to not only contain BMPs, but also to efficiently release them when necessary [16]. Numerous studies have reported on the performance of DDM as a scaffold for BMPs; the osteoconductive and osteoinductive abilities of DDM are deemed to be its greatest strengths. Moreover, DDM, being biocompatible, does not induce foreign body reactions [22,23,24,25]. It has been shown that a biomaterial matrix is required to act as a scaffold for rhBMP-2, to attain maximal efficacy. DDM has adequate porosity to allow cell and blood vessel infiltration, appropriate mechanical stability to withstand compression and tension, biocompatibility, biodegradability, amenability to sterilization, adhesiveness to adjacent bone, affinity for BMPs, and the ability to retain the protein for a sufficient period so as to augment the repair process. The main role of the delivery system for rhBMP-2 is to retain the factor at the site for a prolonged period of time [26,27,28,29]. Ike et al. reported that exogenous rhBMP-2 adsorbed into partly-demineralized dentin from a pulverized partly-demineralized root (allogenic PDM/rhBMP-2) dose-dependently induced heterotopic bone formation, and that PDM provided the minerals and the matrix for adsorption of rhBMP-2, proving that allogenic PDM can be as osteoinductive as autogenous bone [22]. Murata demonstrated that human DDM particles are osteoinductive, insoluble collagenous matrices, and DDM might be an effective rhBMP-2 carrier for bone engineering [23]. Kim et al. reported that human DDM powder might be an effective scaffold for rhBMP-2, as it displayed the highest released-to-loaded rhBMP-2 ratio, the lowest release speed, and the highest expression to induce osteonectin expression, resulting in augmented mature bone formation [16].

In our animal experiment, the rate of new bone formation was 23.8% at two weeks, 45% at eight weeks, and 48% at 12 weeks after placement of human DDM fixed with rhBMP-2. Although the amount of new bone formation induced by human DDM fixed with rhBMP-2 was less than that induced by autogenous bone grafts, it was still higher than that induced by freeze dried bone allograft (FDBA) + platelet-rich growth factor (PRGF) and FDBA within the same amount of time, as observed in the results of previous studies [30]. As the grafted DDM was resorbed, it was replaced with new bone, and so it is thought to be capable of excellent osteoinductive and osteoconductive healing. In the beagle experiment, because the grafted bone mat is thought to take 12 weeks to mature, 48% new bone formation after 12 weeks implies that approximately half the graft area has been replaced with mature bone. This high level of bone healing suggests that DDM can perform the role of an ideal scaffold for rhBMP-2. In other words, after grafting, osteoinductive healing occurs as the rhBMP-2 in the DDM is slowly released, meaning that the DDM itself is osteoinductive and has osteoinductive healing potential.

In the clinical study, ADDM fixed with rhBMP-2 was placed in 23 patients along with 36 implants, and all but one bone graft site were stable and well-maintained without any complications. Tissue samples collected from the maxilla at four months after bone grafting showed dense fibrous connective tissues and angiogenesis around the newly-formed bone and satisfactory bone recovery. The excellent primary and secondary stability seen after implant placement suggests that ADDM fixed with rhBMP-2 produced good healing of the peri-implant bone defect. Histomorphometric analysis of some specimens confirmed that osteogenesis was occurring successfully.

In conclusion, our study confirmed that DDM fixed with rhBMP-2 can induce satisfactory bone recovery without causing any complications in humans. DDM-containing growth factors, such as rhBMP-2, are viable alternatives to autogenous bone graft.

## Figures and Tables

**Table 1:** Clinical findings of the installed implants.

	F/U Period (Months)	Primary Stability (ISQ)	Secondary Stability (ISQ)	Marginal Bone Loss (mm)
Minimum	3.1	49	45	0
Maximum	17.8	85	87	0.8
Average	10.5	71.8	78.0	0.4

F/U: Follow up, ISQ: Implant stability quotient.

## Supplementary Materials

The following are available online at www.mdpi.com/1996-1944/10/9/1049/s1, Figure S1: Histological analysis of new bone formation at the bone defect area after 2 weeks, by hematoxylin/eosin staining (Magnification, A,D: ×12.5, B,E: ×40, C,F: ×100). Figure S2: Histological analysis of new bone formation at the bone defect area after 12 weeks, by hematoxylin/eosin staining (Magnification, A,D: ×12.5, B,E: ×40, C,F: ×100). Table S1: Patient characteristics.

## References

[1] Kim Y.K. (2012). Bone graft material using teeth. J. Korean Assoc. Oral Maxillofac. Surg..

[2] Lee S.H. (2006). Low Crystalline hydroxyl carbonate apatite. J. Korean Dent. Assoc..

[3] Bessho K., Tanaka N., Matsumoto J., Tagawa T., Murata M. (1991). Human dentin-matrix-derived bone morphogenetic protein. J. Dent. Res..

[4] Kim S.G., Kim H.K., Lim S.C. (2001). Combined implantation of particulate dentine, plaster of Paris, and a bone xenograft (Bio-Oss) for bone regeneration in rats. J. Craniomaxillofac. Surg..

[5] Kim Y.K., Kim S.G., Byeon J.H., Lee H.J., Um I.U., Lim S.C., Kim S.Y. (2010). Development of a novel bone grafting material using autogenous teeth. Oral Surg. Oral Med. Oral Pathol. Oral Radiol. Endodontol..

[6] Lee J.Y., Kim Y.K., Yi Y.J., Choi J.H. (2013). Clinical evaluation of ridge augmentation using autogenous tooth bone graft material: Case series study. J. Korean Assoc. Oral Maxillofac. Surg..

[7] Kim Y.K., Yun P.Y., Um I.W., Lee H.J., Yi Y.J., Bae J.H., Lee J. (2014). Alveolar ridge preservation of an extraction socket using autogenous tooth bone graft material for implant site development: Prospective case series. J. Adv. Prosthodont..

[8] Jun S.H., Ahn J.S., Lee J.I., Ahn K.J., Yun P.Y., Kim Y.K. (2014). A prospective study on the effectiveness of newly developed autogenous tooth bone graft material for sinus bone graft procedure. J. Adv. Prosthodont..

[9] Hwang S.T., Han I.H., Huh J.B., Kang J.K., Ryu J.J. (2011). Review of the developmental trend of implant surface modification using organic biomaterials. J. Korean Acad. Prosthodont..

[10] Cheng H., Jiang W., Phillips F.M., Haydon R.C., Peng Y., Zhou L., Luu H.H., An N., Breyer B., Vanichakarn P. (2003). Osteogenic activity of the fourteen types of human bone morphogenetic proteins (BMPs). J. Bone Jt. Surg. Am..

[11] Boyne P.J., Marx R.E., Nevins M., Triplett G., Lazaro E., Lilly L.C., Alder M., Nummikoski P. (1997). A feasibility study evaluating rhBMP-2/absorbable collagen sponge for maxillary sinus floor augmentation. Int. J. Periodont. Restor. Dent..

[12] Hanisch O., Tatakis D.N., Boskovic M.M., Rohrer M.D., Wikesjö U.M. (1997). Bone formation and reosseointegration in peri-implantitis defects following surgical implantation of rhBMP-2. Int. J. Oral Maxillofac. Implants.

[13] Howell T.H., Fiorellini J., Jones A., Alder M., Nummikoski P., Lazaro M., Lilly L., Cochran D. (1997). A feasibility study evaluating rhBMP-2/absorbable collagen sponge device for local alveolar ridge preservation or augmentation. Int. J. Periodont. Restor. Dent..

[14] Sigurdsson T.J., Nygaard L., Tatakis D.N., Fu E., Turek T.J., Jin L., Wozney J.M., Wikesjö U.M. (1996). Periodontal repair in dogs: Evaluation of rhBMP-2 carriers. Int. J. Periodont. Restor. Dent..

[15] Ahn K.J., Park J.C., Kim Y.K. (2014). Experimental study on healing procedure after combined grafting of recombinant human bone morphogenetic protein-2 and anorganic bovine bone. Oral Biol. Res..

[16] Kim Y.K., Um I.W., An H.J., Kim K.W., Hong K.S., Murata M. (2014). Effects of demineralized dentin matrix used as an rhBMP-2 carrier for bone regeneration. J. Hard Tissue Biol..

[17] Nam J.H., Park J.C., Yu S.B., Chung Y.I., Tae G.Y., Kim J.J. (2009). Bone regeneration with MMP sensitive hyaluronic acid-based hydrogel, rhBMP-2 and nanoparticles in rat calvarial critical size defect (CSD) model. J. Korean Assoc. Oral Maxillofac. Surg..

[18] Kim S.J., Kim M.R., Oh J.S., Han I., Shin S.W. (2009). Effects of polycaprolactone-tricalcium phosphate, recombinant human bone morphogenetic protein-2 and dog mesenchymal stem cells on bone formation: Pilot study in dogs. Yonsei Med. J..

[19] Lee J.H., Kim C.S., Choi K.H., Jung U.W., Yun J.H., Choi S.H. (2010). The induction of bone formation in rat calvarial defects and subcutaneous tissues by recombinant human BMP-2, produced in *Escherichia coli*. Biomaterials.

[20] Barr T., McNamara A.J., Sándor G.K., Clokie C.M., Peel S.A. (2010). Comparison of the osteoinductivity of bioimplants containing recombinant human bone morphogenetic proteins 2 (Infuse) and 7 (OP-1). Oral Surg. Oral Med. Oral Pathol. Oral Radiol.Endodontol..

[21] Quinlan E., Thompson E.M., Matsiko A., O’Brien F.J., López-Noriega A. (2015). Long-term controlled delivery of rhBMP-2 from collagen-hydroxyapatite scaffolds for superior bone tissue regeneration. J Control. Release.

[22] Ike K., Urist M.R. (1998). Recycled dentin root matrix for a carrier of recombinant human bone morphogenetic protein. J. Oral Implantol..

[23] Murata M. (2005). Bone engineering using human demineralized dentin matrix and recombinant human BMP-2. J. Hard Tissue Biol..

[24] Murata M., Sato D., Hino J., Akazawa T., Tazaki J., Ito K., Arisue M. (2012). Acid-insoluble human dentin as carrier material for recombinant human BMP-2. J. Biomed. Mater. Res. A.

[25] Murata M., Akazawa T., Hino J., Tazaki J., Ito K., Arisue M. (2011). Biochemical and histo-morphometrical analyses of bone and cartilage induced by human decalcified dentin matrix and BMP-2. Oral Biol. Res..

[26] Burg K.J., Porter S., Kellam J.F. (2000). Biomaterial developments for bone tissue engineering. Biomaterials.

[27] Kirker-Head C.A. (2000). Potential applications and delivery strategies for bone morphogenetic proteins. Adv. Drug Deliv. Rev..

[28] Li R.H., Wozney J.M. (2001). Delivering on the promise of bone morphogenetic proteins. Trends Biotechnol..

[29] Li J., Yang J., Zhong X., He F., Wu X., Shen G. (2013). Demineralized dentin matrix composite collagen material for bone tissue regeneration. J Biomater. Sci. Polym. Ed..

[30] Samandari M.H., Haghighat A., Torabinia N., Taghian M., Sadri L., Naemy V. (2016). Socket preservation using freeze-dried bone allograft with and without plasma rich in growth factors in dogs. Dent. Res. J..

