# Supplementary material for: Evaluation of the Healing Potential of Demineralized Dentin Matrix Fixed with Recombinant Human Bone Morphogenetic Protein-2 in Bone Grafts
Source: Materials (Basel). 2017 Sep 7;10(9):1049. doi: 10.3390/ma10091049 (PMC5615704; doi:10.3390/ma10091049)
Supplement: Supplementary file 1 [file materials-10-01049-s001.pdf]

Table S1. Patient characteristics

| Patient | Age | Sex | Location (#) | Bone graft | Implant placement | Brand name of the implant |
|---|---|---|---|---|---|---|
| 1 | 68 | M | 46 | GBR | Simultaneous | Superline |
| 2 | 53 | F | 14<br>46 | GBR<br>GBR | Simultaneous<br>Simultaneous | CMI<br>CMI |
| 3 | 71 | M | 17 | Sinus graft | Simultaneous | TS III CA |
| 4 | 47 | M | 47 | GBR | Simultaneous | Superline |
| 5 | 56 | M | 13<br>14<br>25<br>27<br>45 | GBR, ridge augmentation<br>GBR, ridge augmentation<br>GBR, ridge augmentation<br>GBR, ridge augmentation<br>GBR, ridge augmentation | Delayed<br>Delayed<br>Simultaneous<br>Simultaneous<br>Simultaneous | TS III CA<br>TS III CA<br>TS III CA<br>TS III CA<br>TS III CA |
| 6 | 54 | F | 44<br>45 | GBR<br>GBR | Simultaneous<br>Simultaneous | Superline<br>Superline |
| 7 | 58 | M | 37 | GBR | Simultaneous | Superline |
| 8 | 59 | M | 16<br>46 | Sinus graft<br>GBR | Simultaneous<br>Simultaneous | Implantium II<br>Implantium II |
| 9 | 50 | F | 36 | GBR | Simultaneous | Implantium II |
| 10 | 39 | M | 15<br>16 | Sinus graft<br>Sinus graft | Simultaneous<br>Simultaneous | TS III HA<br>TS III HA |
| 11 | 73 | M | 16 | GBR | Simultaneous | TS III CA |
| 12 | 40 | F | 17<br>36<br>46<br>47 | GBR<br>GBR<br>GBR<br>GBR | Simultaneous<br>Simultaneous<br>Simultaneous<br>Simultaneous | TS III CA<br>TS III CA<br>TS III CA<br>TS III CA |
| 13 | 32 | M | 36 | GBR | Simultaneous | Superline |
| 14 | 72 | M | 22 | GBR | Simultaneous | Implantium II |
| 15 | 58 | M | 32 | GBR | Simultaneous | MS |
| 16 | 63 | F | 47 | GBR | Simultaneous | Superline |
| 17 | 68 | M | 26 | Sinus graft | Simultaneous | TS III CA |
| 18 | 75 | F | 34 | GBR | Simultaneous | CMI |
| 19 | 82 | M | 45<br>46 | GBR<br>GBR | Simultaneous<br>Simultaneous | Superline<br>Superline |
| 20 | 59 | M | 47 | GBR | Simultaneous | TS III CA |
| 21 | 45 | M | 36 | GBR | Simultaneous | Implantium II |
| 22 | 64 | M | 26 | Sinus graft | Delayed | CMI |
| 23 | 77 | F | 35<br>36 | GBR | Simultaneous<br>Simultaneous | Superline<br>Superline |

Location(#): Tooth number, Superline, Implantium II (Dentium, Suwon, Korea), TS III CA, TS III HA, MS (Osstem Implant Co., Busan, Korea), CMI (Neobiotec, Seoul, Korea)

Supplement

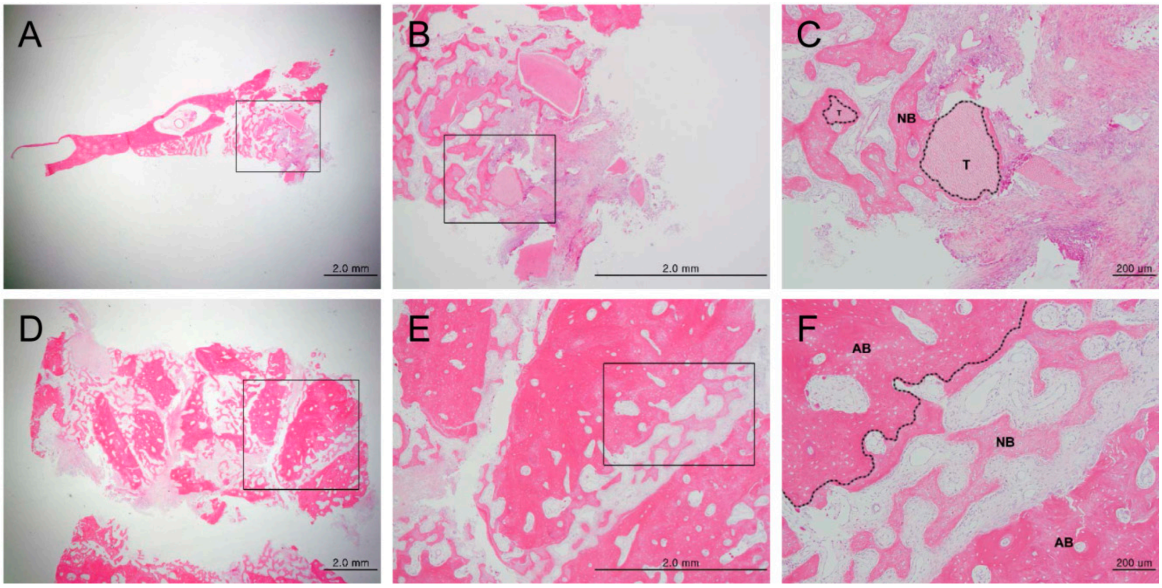


**Figure S1.** Histological analysis of new bone formation at the bone defect area after 2 weeks, by hematoxylin/eosin staining (Magnification, A,D: ×12.5, B,E: ×40, C,F: ×100). A, B, C: experimental group (DDM fixed with rhBMP-2), D, E, F: control group (autogenous bone graft). Figures B and E are higher magnification views of the figures A and D, respectively. Figures C and F are higher magnification views of the figures B and E, respectively. C: The area enclosed within dotted line represents DDM fixed with rhBMP-2, and newly formed bone is observed around the DDM. F: The area enclosed within the dotted line indicates autogenous bone graft, and newly formed bone was found around the autogenous bone graft. Scale bars, 2.0 mm (A, B, D, E), 200 µm (D, F). T: DDM fixed with rhBMP-2, NB: new bone, AB: autogenous bone.

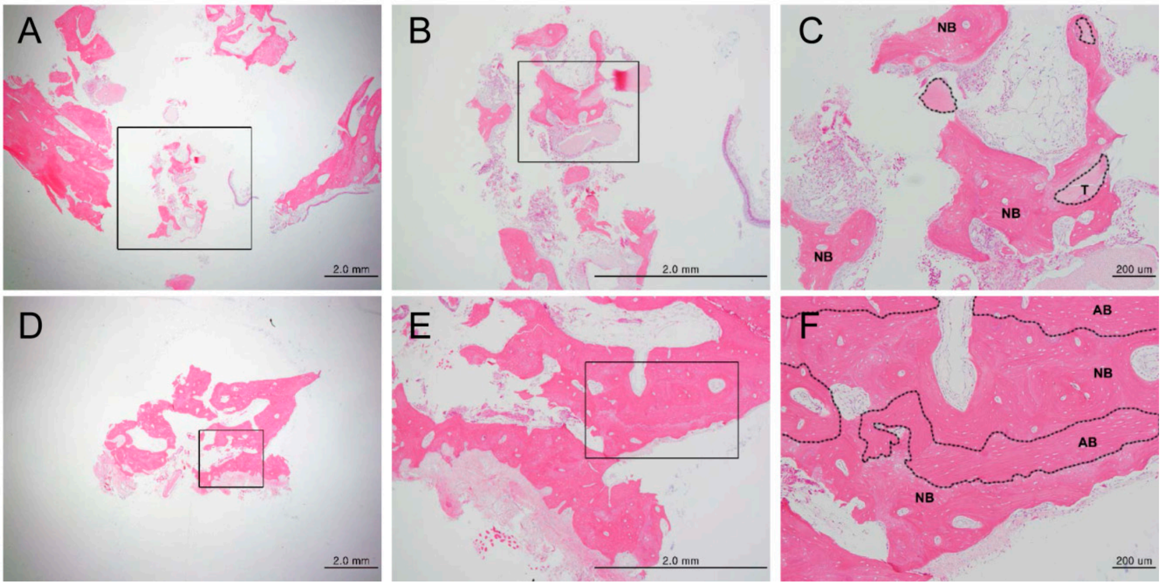


**Figure S2.** Histological analysis of new bone formation at the bone defect area after 12 weeks, by hematoxylin/eosin staining (Magnification, A,D: ×12.5, B,E: ×40, C,F: ×100). A, B, C: experimental group (DDM fixed with rhBMP-2), D, E, F: control group (autogenous bone graft). Figures B and E are higher magnification views of the figures A and D, respectively. Figures C and F are higher magnification views of the figures B and E, respectively. C: The area enclosed within the dotted line represents DDM fixed with rhBMP-2, and newly formed bone seen around the DDM. F: The area within the dotted line indicates autogenous bone graft, and newly formed bone found around autogenous bone graft. Scale bars, 2.0 mm (A, B, D, E), 200 µm (C, F). T: DDM fixed with rhBMP-2, NB: new bone, AB: autogenous bone.
